# Successful Advanced Maternal Age Pregnancy with Mosaic Turner Syndrome Conceived after Ovulation Induction with Clomiphene Citrate: A Case Report

**DOI:** 10.1155/2014/934740

**Published:** 2014-06-11

**Authors:** Masahiro Murakami, Kenji Hinokio, Machiko Kiyokawa, Mikio Morine, Takeshi Iwasa

**Affiliations:** ^1^Department of Obstetrics and Gynecology, Shikoku Medical Center for Children and Adults, Senyu-cho 2-1-1, Zentsuji City, Kagawa 765-8507, Japan; ^2^Department of Obstetrics and Gynecology, Institute of Health Biosciences, The University of Tokushima Graduate School, 3-18-15 Kuramoto-cho, Tokushima 770-8503, Japan

## Abstract

Turner women typically experience gonadal dysfunction that results in amenorrhea and sterility. We encountered a case of mosaic Turner syndrome where conception was possible after ovulation induction with clomiphene citrate (CC). The patient's ovaries were overresponsive to induction with CC. The challenges and successful outcome are reported.

## 1. Case Presentation

Recently, we encountered a case of mosaic Turner syndrome (TS) conceived after ovulation induction with clomiphene citrate (CC). The patient was found to have a mosaic chromosome complement (45,X [14]/46,XX [16]), when she was 12 years old. She married at 27 years old. There was no abnormality in the result of cardiovascular ultrasound examination, bone density scan, and the levels of serum thyroid hormone. She wished for a child during the subsequent eight years. Unfortunately, she did not become pregnant. Then, she hoped to receive an examination about preserving fertility. When the patient was referred to our hospital for her primary sterility, she was a 35-year-old Japanese woman who was 38 kg and 138 cm. She was not found with webbed neck and cubitus valgus. The vaginal ultrasound showed that the size of uterus was 72 × 27 mm. In the follicular phase, luteinizing hormone (LH) was 4.9 mIU/mL and follicular stimulating hormone (FSH) was 3.5 mIU/mL. In the luteal phase, the level of progesterone was 15.8 ng/mL and the ultrasound finding of endometrial thickness was 8.5 mm. A sperm analysis was not abnormal. CC is initiated at the dose of 50 mg daily for 5 days. We canceled first cycle with multiple follicles due to the increased risk of a higher order multiple pregnancy. However, she had been pregnant during washout periods; ultrasound findings showed mass with multivesicular pattern in her uterus. Moreover, human chorionic gonadotropin (hCG) was 52700 mIU/mL. This was diagnosed as hydatidiform mole. We confirmed the negative beta hCG result after procedure with dilatation and curettage. She was given in 50 mg daily dose for 5 days, again. She was pregnant with only one cycle of an ovulation induction. Unfortunately, she took the result in the abortion of her pregnancy during her first trimester. Moreover, her ovaries were extremely sensitive to treatment with CC. The dose in subsequent cycles was reduced to 25 mg/day. In the subsequent cycle, the single follicle was developed. After eighth cycles of ovulation induction with CC 25 mg, she had become pregnant at 38 years. Nuchal translucency screening was negative at 11 weeks and 2 days. She did not want to undergo the amniocentesis after physician's explanation. The newborn female baby (birth weight 2628 g) was delivered by cesarean section at 38th week of gestation due to cephalopelvic disproportion. Major physical anomaly was not found. Chromosomal analysis revealed a 46,XX karyotype.

## 2. Discussion

Turner women typically experience gonadal dysfunction, which results in amenorrhea and sterility. It was reported that sufficient follicles survive 15% of TS and approximately 2% achieve spontaneous pregnancies, so TS has premature menopose [1–3]. It is reported that some women have successful pregnancies using donated eggs. In addition, there is a higher incidence of miscarriages and fetal death in spontaneous pregnancies than in the pregnancy following the eggs donation among Turner women [2, 3].

The domestic PCOS criteria of the Japanese Society of Obstetrics and Gynecology (JSOG) consist of the association of all three of the following factors: chronic anovulation, LH hypersecretion and/or hyperandrogenism, and the presence of polycystic ovaries (PCOS). PCOS are treated with induction of ovulation with CC or gonadotropin administration. It is well known that there is the risk of overresponse with multiple follicular development and ovarian hyperstimulation in women with PCOS. This etiology is not clear. Multiple pregnancies involve a greater risk of both maternal and fetal complications than singleton pregnancies [4]. If there are more than two follicles due to CC 50 mg/day, the dose in subsequent cycles can be reduced to 25 mg/day in the PCOS patients [5]. However it is the absent of polycystic ovaries in this case, it was the presence of ovulatory disorder, anovulation, and LH hypersecretion. In addition, multiple follocules developed following CC treatment. Therefore we decided to reduce the dose of CC by the method as a patient of PCOS.

The egg donation is not admitted in Japan society. It might be better to start the reproductive technique in an early stage. However, the prevention of multiple pregnancies is important in assisted reproduction of TS.
